# An In-Field Assessment of the P.ALP Device in Four Different Real Working Conditions: A Performance Evaluation in Particulate Matter Monitoring

**DOI:** 10.3390/toxics12040233

**Published:** 2024-03-22

**Authors:** Giacomo Fanti, Francesca Borghi, Davide Campagnolo, Sabrina Rovelli, Alessio Carminati, Carolina Zellino, Andrea Cattaneo, Emanuele Cauda, Andrea Spinazzè, Domenico Maria Cavallo

**Affiliations:** 1 Department of Science and High Technology, University of Insubria, 22100 Como, Italy; davide.campagnolo@uninsubria.it (D.C.); sabrina.rovelli@uninsubria.it (S.R.); acarminati@uninsubria.it (A.C.); czellino@uninsubria.it (C.Z.); andrea.cattaneo@uninsubria.it (A.C.); andrea.spinazze@uninsubria.it (A.S.); domenico.cavallo@uninsubria.it (D.M.C.); 2 Department of Medical and Surgical Sciences, University of Bologna, 40126 Bologna, Italy; francesca.borghi12@unibo.it; 3 Center for Direct Reading and Sensor Technologies, National Institute for Occupational Safety and Health, Pittsburgh, PA 15236, USA; cuu5@cdc.gov; 4 Centers for Disease Control and Prevention, Pittsburgh, PA 15236, USA

**Keywords:** miniaturized monitors, microenvironment, air quality, air pollution, exposure assessment, low-cost monitor

## Abstract

This study aimed to assess the performance, in terms of precision and accuracy, of a prototype (called “P.ALP”—Ph.D. Air Quality Low-cost Project) developed for monitoring PM_2.5_ concentration levels. Four prototypes were co-located with reference instrumentation in four different microenvironments simulating real-world and working conditions, namely (i) office, (ii) home, (iii) outdoor, and (iv) occupational environments. The devices were evaluated for a total of 20 monitoring days (approximately 168 h) under a wide range of PM_2.5_ concentrations. The performances of the prototypes (based on the light-scattering working principle) were tested through different statistical methods. After the data acquisition and data cleaning processes, a linear regression analysis was performed to assess the precision (by comparing all possible pairs of devices) and the accuracy (by comparing the prototypes against the reference instrumentation) of the P.ALP. Moreover, the United States Environmental Protection Agency (US EPA) criteria were applied to assess the possible usage of this instrumentation, and to evaluate the eventual error trends of the P.ALP in the data storage process, Bland–Altman plots were also adopted. The outcomes of this study underlined that the P.ALP performed differently depending on the microenvironment in which it was tested and, consequently, on the PM_2.5_ concentrations. The device can monitor PM_2.5_ variations with acceptable results, but the performance cannot be considered satisfactory at extremely low and remarkably high PM_2.5_ concentrations. Thanks to modular components and open-source software, the tested device has the potential to be customized and adapted to better fit specific study design needs, but it must be implemented with ad hoc calibration factors depending on the application before being used in field.

## 1. Introduction

### 1.1. Background and Problem Statement

There is a high demand for routine air quality assessment globally, and this is generally due to a growing population that is especially concentrated in urban areas and an increased and evolving awareness of the risks associated with air pollution [1]. Concerning air pollution, a prominent problem is fine airborne particulate matter (PM), which is produced by both natural and anthropogenic processes [2]. Exposure to fine PM represents a significant risk factor for human health because this pollutant can reach deep regions of the respiratory system and has the potential to cause different health effects, such as cardiovascular disease, asthma, and decreased lung functions [3,4]. Providing high-resolution data, both in terms of spatial and temporal resolution, regarding the monitoring of airborne pollutants is fundamental for an efficient implementation of air quality guidelines and policies. High-resolution monitoring could supplement data from air quality monitoring stations that are used to assess the ambient air quality as defined in Europe in the Directive 2008/50/EC [5]. The development and availability of new monitoring technologies in the past few decades have opened several new possibilities and applications in air quality monitoring, mainly thanks to their characteristics of good portability, user-friendliness, low power consumption, high data storage capability, and relatively low cost. Integrating modern technologies and reference-grade techniques could improve the data provided by air quality monitoring systems to a higher level of spatial coverage while maintaining precision and accuracy as much as possible [6,7,8]. The number of low-cost monitoring devices, defined in previous publications [9,10] as “Next Generation Monitor and Sensors” (NGMSs), that are currently available is rising [9,10]. This increased availability of NGMSs can lead to the adoption of air quality monitoring of airborne pollutants in occupational settings [9] and for indoor air quality studies [11]. However, the potential use of NGMSs is also associated with questions and concerns regarding their applications in the field, their performances, and the reliability of the data provided. Improvements are needed to further enhance the performance of these technologies and allow them to play a primary role in the field of exposure assessment [10]. Several manuscripts focused on the assessment of the performances of these devices have been published, pairing them to reference-grade instrumentation and mostly evaluating their precision and accuracy [12,13,14,15,16]. The assessment of the performances of these emerging technologies must be conducted before the adoption of the abovementioned devices, even if the US National Institute for Occupational Safety and Health (NIOSH) approach “Right Sensors used Right” highlighted that these devices could be merged with reference-grade instrumentation to understand their best applicability for use in the field [17]. This latter approach aims to promote the competent development, adoption, and interpretation of real-time monitors and direct reading methodologies. Moreover, it aims to improve the interpretation of the data to take action in work environments.

### 1.2. Aim of the Study

As described in a previous publication [18], the P.ALP (Ph.D. Air Quality Low-cost Project) is a prototype for monitoring the concentration of PM_2.5_, and it was conceptualized, implemented, and created at the University of Insubria (Como, Italy) with the intention of solving some of the issues experienced in past research studies [19,20,21], specifically portability, user-friendliness, power consumption, data storage capability, and high cost. The P.ALP was tested in a calm-air aerosol chamber, and the results of these tests show that the P.ALP can follow PM_2.5_ concentrations trends with reasonable accuracy, but its performance needs to be improved through calibration factors [22]. In the present study, the device was evaluated in the field to explore its performance in non-controlled contests in four different microenvironments, aiming to investigate real working conditions and a wide range of PM_2.5_ concentrations. The selected microenvironments were (i) office, (ii) home, (iii) outdoor, and (iv) occupational environments. This study is focused on the evaluation of the P.ALP’s performance in terms of precision and accuracy [20,23] compared to the reference-grade instrumentation adopted. A further goal of this research is to find the best application field for the P.ALP device by adopting the criteria suggested by the US EPA—United States Environmental Protection Agency [16].

## 2. Material and Methods

### 2.1. Instrumentation and Setup

The P.ALP, in its configuration at the time of the data acquisition campaign, can acquire data on (i) airborne particulate matter, also called PM concentrations (PM_1_, PM_2.5_, and PM_10_); (ii) temperature (T); and (iii) relative humidity (RH). The PM data were generated and acquired using a PMS5003 sensor (Plantower, Nanchang, China), a particle concentration sensor based on the light scattering principle, which can be used to obtain the number of airborne particles in a volume of air and to generate an output via a digital interface, providing mass concentration data over time [18]. Temperature and relative humidity data are obtained using the DHT22 sensor (AZ-Delivery Vertriebs GmbH, Deggendorf, Germany) (also called AM2302), which uses a capacitive humidity sensor and a thermistor to measure the surrounding air [18]. The P.ALP device can be powered by any commercial power bank (e.g., a 10,000 mAh battery can supply the device with power for at least 48 h), and the collected data are stored on an onboard MicroSD card. Four units, hereafter called P.ALP_0, P.ALP_1, P.ALP_2, and P.ALP_3, were assembled with identical characteristics and configurations to evaluate the precision of the P.ALP devices. The reference data for time-weighted PM_2.5_ concentrations, which are used to evaluate the P.ALP devices’ performances, were acquired through a reference-grade gravimetrical sampler (i.e., Harvard Impactor—HI) operated at a flow rate of 10 L/min, which provides a 50% cut-off point at 2.5 µm (collection substrate: PTFE w/PMP ring; diameter: 37 mm; porosity: 2 µm) [24]. The time-weighted PM_2.5_ concentration data were also used, for each sampling period, to calculate a correction factor for the 1 min resolution data points obtained using a non-reference-grade direct reading instrument, Aerocet 831-Met One Instrument Inc., Grant Pass, OR, USA. The Aerocet-831 device was co-located with the above-mentioned instrumentation to obtain data characterized by a high temporal resolution, which were used as a reference for the P.ALP’s performance evaluation.

### 2.2. Data Collection

The data were collected through 20 monitoring days, divided into 4 different microenvironments (office, home, outdoor, and occupational) for a total of approximately 168 h of monitoring divided into five 8 h sessions per microenvironment. The first five days of monitoring, which were conducted at the University of Insubria Offices (Via Valleggio 11—Como, Italy), investigated an indoor environment in which low concentrations of PM_2.5_ were expected. From day 6 to day 10, the instruments were placed in a domestic environment, indoors, in a residential area (Villa Guardia—Como, Italy). The third phase of the study was conducted at a previously selected outdoor site at the University of Insubria (Via Valleggio, 11—Como, Italy), representative of an urban background area [25]. The last five monitoring days were performed in an occupational environment at the production site of a rubber molding factory; the monitoring sessions in this microenvironment lasted only 6 h to avoid overloading the reference samples. All of the data acquired using the instruments were downloaded at the end of each monitoring session. In addition, the HI was cleaned, and the sampled filter was substituted by a new one after each session. The weighing filters were conditioned in a controlled environment (T: 20.0 ± 1 °C; RH: 50 ± 5%) for at least 24 h. The filters were weighed before and after sampling with a microbalance tool (Gibertini Micro1000, Novate, Milan, Italy; readability: 1 µg). An electrical C-shaped ionizer (HAUG GmbH & Co. KG, Leinfelden-Echterdingen, Germany) was used to avoid electrostatic charges from the surface of the filter. Two laboratory blanks were weighed under the abovementioned conditions to identify any possible anomalies in the weighing room environment (T and RH variations). The accuracy of the microbalance was checked through certified masses of 1 g and 100 mg, which were weighed at the beginning and at the end of each weighing session, allowing for deviations of ≤3 and 5 µg, respectively, from the true value.

### 2.3. LOD and LOQ

The P.ALP’s limit of detection (LOD) and the limit of quantification (LOQ) calculations were performed based on data acquired under controlled conditions using a calm air dust chamber [26] in a previous laboratory study [22]. The Kaiser and Specker (1956) method [27] was used to calculate the LOD (2.12 µg/m^3^) of the P.ALP, and the LOQ was calculated as being equal to three times the LOD (6.36 µg/m^3^), as reported by the Reg. (EU) 333/2007 [Eq. D] [28]. Initially, for the study reported in this manuscript, the values lower than the LOD were maintained in the database, and a first descriptive statistic was conducted (Table S1 and Figure S6). Considering the comparable data distribution between the P.ALPs and the Aerocet-831 device at the very low PM_2.5_ concentrations (Figure S6), the authors decided to remove the data that were lower than the P.ALP’s LOD and to focus the statistical analysis of this manuscript on the PM_2.5_ concentrations higher than 2.12 µg/m^3^. This decision was taken along with the conviction that the application field of the P.ALP is not the characterization of extremely low PM_2.5_ exposure conditions, but rather the characterization of scenarios in which high concentrations or significant changes in concentrations of PM_2.5_ are expected.

### 2.4. Data Treatment and Statistical Analysis

After each monitoring session, raw data collected by the P.ALPs were downloaded and organized in a dedicated database. Unrealistic data due to instrument malfunctions were identified and removed from the database. To exclude these data, the concentration distributions were truncated above the 99th percentile and below the 1st percentile. The 33rd and 66th percentiles (6.69 µg/m^3^ and 30.87 µg/m^3^, respectively) were calculated on the whole dataset to divide the database into three different concentration ranges (namely low concentrations (LOD < x < 6.69 µg/m^3^), medium concentrations (6.69 < x < 30.87 µg/m^3^), and high concentrations (x > 30.87 µg/m^3^)) to investigate the performance metrics of the prototypes in each concentration range. By session averaging the data acquired using the Aerocet device, the data acquired using the HI were used to calculate specific correction factors, one for each microenvironment (1.62, 1.65, 0.91, and 0.83, respectively, for office, home, outdoor, and occupational microenvironments), to correct the values acquired using the Aerocet device (corrected Aerocet value = raw value × correction factor) and to be able to consider it as a reference value [19,20] for a PM_2.5_ with a frequency of 1 datapoint a minute. All of the data reported in this manuscript, acquired using the Aerocet device, were already corrected by the application of the abovementioned correction factors. The latter were used as high time-resolution reference data to evaluate the P.ALP. Descriptive statistics were calculated for PM_2.5_ concentration outcomes for (i) the four P.ALPs, (ii) the reference instrument (Aerocet), (iii) T, and (iv) the acquired data on the RH. To explore the data distribution, a Kolmogorov–Smirnov test was performed on the whole dataset, and the result was of a non-normal (or non-log-normal) distribution. Consequently, a non-parametric Mann–Whitney test was performed, concerning all of the possible pairings of the Aerocet device and P.ALPs, as the first analysis to assess the differences between two independent groups of a continuous variable. A *p*-value lower than 0.05 was considered statistically significant for all tests. The evaluation of the performances of the P.ALPs was carried out through different tests as follows: (i) By using a linear regression analysis, the precision of the devices was assessed following the indications published by Watson et al., 1998 [23], a comparability analysis was performed for each pair of tested devices, and the predictability of one P.ALP compared to that of another one was determined. (ii) The accuracy of the prototypes was evaluated using the same approach but considering the reference measurement (Aerocet) as the independent variable and the investigated devices (the four P.ALPs) as the dependent variable. Using this approach, two measurement techniques can be considered comparable if the correlation coefficient (R) exceeds the value of 0.9, and they can be considered reliably predictive of each other if the previous criteria meet the following two criteria: (1) the slope (m) equals unity within three standard errors and (2) the intercept (q) does not differ from zero within three standard errors (SEs) [23]. The analysis of precision (comparison between the P.ALPs) and accuracy (comparison between the P.ALPs and the Aerocet device) was conducted on the entire database. Finally, following the US EPA air sensor guidebook [3,16,29], the Coefficient of Variation (CV), and the Mean Normalized Bias (MNB) of the values acquired using the devices were calculated with the aim of placing the P.ALP prototype in its proper application field for PM_2.5_ monitoring. The criteria suggested by Williams et al., 2014 [16], in particular, are the following:

- Tier I: Education and Information (−0.5 < MNB < 0.5 and CV < 0.5);
- Tier II: Hotspot Identification and Characterization (−0.3 < MNB < 0.3 and CV < 0.3);
- Tier III: Supplemental Monitoring (−0.2 < MNB < 0.2 and CV < 0.2);
- Tier IV: Personal Exposure (−0.3 < MNB < 0.3 and CV < 0.3);
- Tier V: Regulatory Monitoring (−0.1 < MNB < 0.1 and CV < 0.1).

The possible error trends of the P.ALPs were assessed using Bland–Altman plots [30]. The graphs were built based on the session average data, and the absolute deviation between the results of the reference instrument (Aerocet) and the compared instrument (P.ALP) was reported for each pair of measurements. The average errors and the relative upper and lower 95% confidence interval (95% CI) were also evaluated. Statistical analysis of the collected data was performed using the SPSS Statistics 20.0 software (IBM, Armonk, NY, USA).

## 3. Results

A total of 20 monitoring sessions (collectively more than 168 h of testing) were performed between February and March 2022. A brief overview of the microenvironment conditions during the testing sessions is reported in Table 1. All of the Aerocet device’s PM_2.5_ data that are presented were already corrected based on the HI data. The relative humidity and temperature data reported in Table 1 were collected using the four P.ALPs and their values were reported; the mean RH% values were not reported because the RH% range, in which the PMS5003 sensor was evaluated to assess it with its nominal functioning RH% range (0~99%), was provided. Furthermore, the T Min. and Max. values were not reported because, due to the study design, their variations during the monitoring sessions were extremely low and not of interest to the aim of this manuscript.

### 3.1. Descriptive Statistics

**Figure 1:**
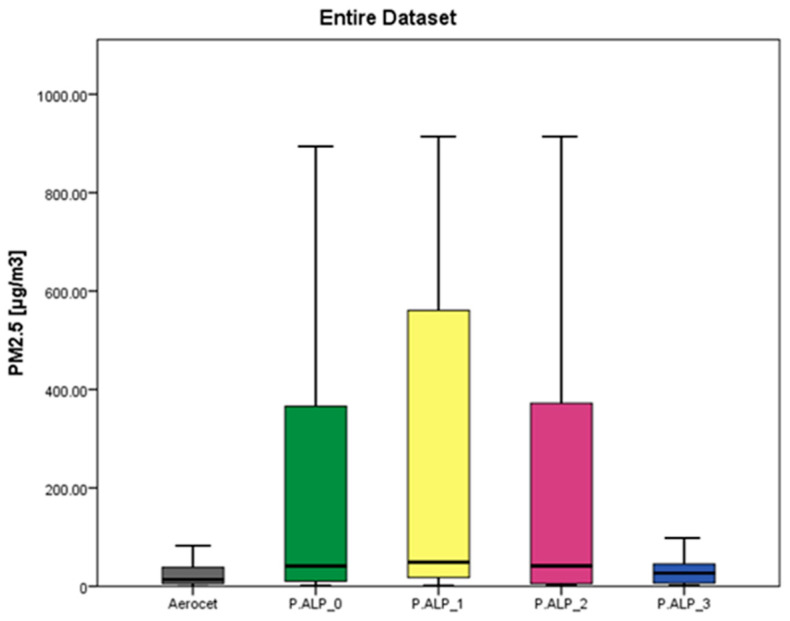
A box plot chart reporting the concentration values, expressed in µg/m^3^, of the reference instrument (Aerocet) and the four P.ALP prototypes considering the complete set of data acquired during the whole study. The central black mark is the median, the edges of the box are the 25th and the 75th percentiles, and the error bars show the extent of the most extreme data points that are not considered outliers.

As mentioned in Section 2.3 of this manuscript, the authors decided to remove the data that were lower than the LOD, which were acquired using the P.ALPs, from the original database so that all of the statistical analyses are focused on values higher than 2.12 µg/m^3^. This decision was made based on the conviction that, even if a considerable portion of the data has been removed, the amount of data analyzed is considered acceptable to properly evaluate the device under investigation. Table 2 and Figure 1 present the summary statistics regarding the PM_2.5_ concentrations, expressed in µg/m^3^, acquired using the four prototypes and the Aerocet device (reference instrument for this study). Summary statistics for the dataset divided based on concentration range (Table S2, Figures S1 and S2) and microenvironment (Table S3, Figures S3–S5) are also available. The data regarding the low concentrations and the office microenvironment are not shown in the figures due to their poor numerosity after the removal of the <LOD data.

### 3.2. Precision

To evaluate the precision of the prototypes, as described in Section 2.4, each pair of co-located P.ALPs was evaluated through a linear regression analysis following Watson et al.’s criteria [23] to state whether they are comparable and mutually predictable with both of the previous prototypes or neither of them (Table 3). Below are the outcomes from the analysis of the entire dataset, but the evaluations were also conducted by splitting the dataset based on the concentration range (Table S2) and microenvironment (Table S3).

As shown in Table 3, after performing this analysis and considering the entire dataset, the R coefficient results are always higher than the 0.9 value (the minimum criteria for the comparability of two devices by Watson and colleagues [23]), so it is possible to consider that the four prototypes are always comparable with each other. On the contrary, except for the comparison between P.ALP_0 and P.ALP_2, the devices never met the minimum criteria to be considered mutually predictable. This behavior does not change significantly when considering different concentration ranges (Table S4) and microenvironments (Table S5).

### 3.3. Accuracy

The evaluation of the accuracy of the P.ALPs was performed by applying Watson et al.’s 1998 guidelines [23]. All of the devices were compared against the data acquired from the co-located reference instrumentation (i.e., Aerocet). The results of the accuracy analysis are reported in Table 4; it is possible to consider that the four prototypes are always comparable, but not mutually predictable, with the reference instrument. However, the P.ALP accuracy changes significantly when considering different concentration ranges (Table S6) and microenvironments (Table S7).

### 3.4. Application Field Based on US EPA Guidelines

The US Environmental Protection Agency’s guidelines [16] were adopted to find an application field for the P.ALP prototypes. Following these latter guidelines, Table 5 presents the results of this analysis for the entire database. The P.ALPs’ performances change significantly when considering different concentration ranges (Table S8) and microenvironments (Table S9).

### 3.5. Error Trends

**Figure 2:**
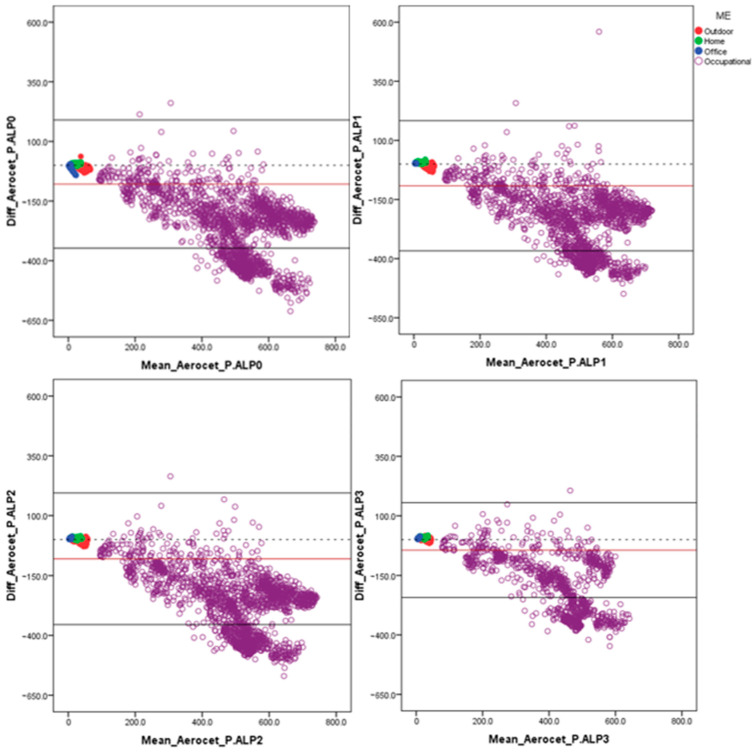
The Bland–Altman plots of the data acquired from the four P.ALPs plotted against the reference instrument (Aerocet); both the X and Y axes are expressed in µg/m^3^. The data referring to the office microenvironment are highlighted in blue, the data referring to the home microenvironment are shown in green, the data referring to the outdoor microenvironment are shown in red, and the data referring to the occupational microenvironment are highlighted in purple. The dotted black line indicates the theoretical perfect agreement between the two compared instruments (P.ALP and Aerocet). The solid red line represents the mean error between the compared techniques, and the two solid black lines represent the upper and lower 95% confidence intervals, respectively.

The Bland–Altman approach [30] was adopted to better understand the possible error trends of the prototypes under investigation. In this subsection, the statistics (Table 6) and the highlights of the analyses conducted while considering the whole dataset are shown (Figure 2), but as carried out for the previous analysis, the data were also investigated by splitting the dataset based on the concentration range and microenvironment in this case. These latter analyses are presented in the Supplementary Materials of this manuscript (Figures S7–S9).

## 4. Discussion

### 4.1. Descriptive Statistics

By considering the outcomes of the analysis of the whole dataset (Figure 1), it can be seen that the mean concentration values (mean ± S.D.) of the P.ALPs differ (P.ALP_0 = 198 ± 289; P.ALP_1 = 237 ± 297; P.ALP_2 = 199 ± 293 and P.ALP_3 = 138 ± 228 µg/m^3^) from the concentration values obtained through the reference instrument (Aerocet = 88 ± 154 µg/m^3^). In fact, as reported in Table S10, the obtained results of the Mann–Whitney analyses showed statistically significant differences between the P.ALPs and the Aerocet device. P.ALP_3 provides the measured values closest to the reference, on average. Moreover, on average, the P.ALPs tend to overestimate the concentration of PM_2.5_ compared to the reference. Considering the outcomes presented in Table 2 (whole dataset), the median and mean values of the reported devices are hugely different from each other, and the SD values are remarkably high, too. These are presumably due to the wide range of PM_2.5_ concentrations investigated in the four microenvironments. In fact, as reported in Table S3, splitting the analyses based on microenvironment highlights remarkably similar means and median values between each other and lower SD values in comparison to the ones reported in Table 2. The results of the PM_2.5_ measurement split based on the microenvironment (Table S3; Figures S3–S5) and concentration range (Table S2; Figures S1 and S2) are presented in the Supplementary Materials of this manuscript. Data regarding the office microenvironment and the low concentrations are not shown as figures due to their poor numerosity after the removal of the data below the LOD. Based on a preliminary qualitative–quantitative comparison, it is possible to observe that P.ALPs have different performances depending on the PM_2.5_ concentration range (Table S2) and the microenvironment in which the measurement was carried out (Table S3). These first data suggest that the best performance of P.ALP is obtained in home and outdoor microenvironments, where it is more likely to find low and medium concentrations.

### 4.2. Precision

Following Watson et al.’s criteria [23], after performing the analysis while considering the entire dataset, it is possible to assert that of all the prototypes were comparable, but only P.ALP_0 and P.ALP_2 resulted in mutually predictable data (Table 3). After splitting the dataset based on concentration range and microenvironment, only the comparisons at medium and high concentrations were carried out because of the poor numerosity of data concerning low concentrations after the data below the LOD were removed. The result of the analysis was generally the same as those obtained with the whole dataset; all of the prototypes showed to be comparable, and only in one case (P.ALP_0 and P.ALP_3 at high concentrations), they were mutually predictable (Table S4). Moreover, after splitting the dataset based on microenvironment, all of the prototypes were comparable to each other, except for P.ALP_0 and P.ALP_1 and P.ALP_1 and P.ALP_3, which were both in the outdoor microenvironment. They were only mutually predictable in four cases (P.ALP_0 and P.ALP_2 in the outdoor microenvironment; P.ALP_1 and P.ALP_2 in the home microenvironment; P.ALP_1 and P.ALP_3 in the occupational microenvironment; and P.ALP_2 and P.ALP_3 in the occupational microenvironment), as shown in Table S5. Overall, although some exceptions highlight some limitations, the prototypes show a satisfactory performance in terms of precision.

### 4.3. Accuracy

When considering the outcomes presented in Table 4 and applying Watson et al.’s criteria [23] through the regression analysis approach, performed on the whole dataset, it is possible to assert a situation of comparability between all four prototypes and the reference instrument, the Aerocet device. Moreover, two prototypes out of four (P.ALP_0 and P.ALP_2) highlight a situation of mutual predictivity between them and the reference device (Aerocet). More analyses were performed to further investigate the accuracy of the prototypes in different situations, and to carry those out, the regression was conducted by splitting the dataset based on concentration range and microenvironment. The outcomes of this latter analysis are reported in Tables S6 and S7. After splitting the dataset based on concentration range, at low concentrations, none of the P.ALPs were comparable to the reference instrument. At the mean concentrations, P.ALP_1 was the only one that was not comparable to the Aerocet device, and at the high concentration, all of the devices were comparable to the reference instrument. Furthermore, none of the P.ALPs were ever classified as being able to predict the Aerocet data, as reported in Table S6. The analysis was also conducted by splitting the dataset based on microenvironment, and the outcomes highlight that, in this case, none of the P.ALPs were comparable to (nor mutually predictive with) the reference instrument in the office and the occupational microenvironments. Concerning the home microenvironment, all of the P.ALPs were comparable but never mutually predictive of the reference instrument. Moreover, concerning the outdoor microenvironment, all of the prototypes were comparable with the reference instrument except for P.ALP_1, and as shown in Table S7, only P.ALP_0 and P.ALP_2 were able to predict the Aerocet data. Overall, the prototypes showed a satisfactory performance in terms of accuracy, but a significantly different performance was observed in the different scenarios investigated and at different concentration levels.

### 4.4. US EPA Guidelines

After applying the US EPA guidelines on the Air Sensors [3,16], it was possible to identify, based on the CV and MNB parameters, an application field of the P.ALP prototype depending on the working conditions in which it was placed. The first analysis was conducted on the entire dataset and, as reported in Table 5, none of the P.ALPs were able to be placed in one of the five tiers suggested by the US EPA criteria. To further investigate the performances of the P.ALPs, the same approach was adopted by splitting the database based on concentration range as follows (Table S8): (i) at low concentrations, P.ALP_1 and P.ALP_3 were placed in Tier I (i.e., for education and information purposes), and P.ALP_2 was placed in Tier II (i.e., for hotspot identification and characterization purposes) and Tier IV (i.e., for personal exposure purposes). (ii) At medium concentrations, P.ALP_0 and P.ALP_3 were placed in Tier II and Tier IV, while P.ALP_1 and P.ALP_2 were placed in the Tier I application field. Furthermore, (iii) at high concentrations, P.ALP_3 was the only one eligible to be placed in a possible application field (i.e., Tier I). The analysis was also conducted by splitting the dataset based on microenvironment (Table S9). Concerning the (i) office microenvironment, P.ALP_1 and P.ALP_2 were placed in the Tier II and Tier IV applicability fields, and P.ALP_3 was placed in Tier I. Regarding (ii) the home microenvironment, P.ALP_0 was placed in Tier II and Tier IV, P.ALP_1 was placed in Tier V (i.e., for regulatory monitoring purposes), and P.ALP_2 and P.ALP_3 were placed in Tier III (i.e., supplemental monitoring). The (iii) outdoor microenvironment highlighted that P.ALP_3 had a good performance, which met the Tier V criteria, compared to P.ALP_0 and P.ALP_2, which only met the Tier I criteria. Regarding the (iv) occupational microenvironment, P.ALP_3 was the only one that was placed in one of the tiers suggested by the US EPA (i.e., Tier I).

### 4.5. Error Trends

Figure 2 and Figures S7–S9 show, through the Bland–Altman plots approach, that all the P.ALPs are affected by the same error trend. It could be stated that the higher the PM_2.5_ concentrations, the higher the overestimation of the devices compared to the data acquired using the reference instrument. As expected, after splitting the data distribution based on microenvironment, it is worth noting that the highest bias between the two techniques compared was found in the occupational microenvironment (P.ALP_0: −260 ± 135 µg/m^3^; P.ALP_1: −247 ± 132 µg/m^3^; P.ALP_2: −269 ± 133 µg/m^3^; P.ALP_3: −188 ± 121 µg/m^3^), where the highest monitored PM_2.5_ concentrations were found. On the contrary, the average error observed in the office and home microenvironments highlights the tendency towards underestimation (P.ALP_0: −3.8 ± 7.8 µg/m^3^; P.ALP_1: 2.5 ± 0.8 µg/m^3^; P.ALP_2: 2.7 ± 1.2 µg/m^3^; P.ALP_3: 3.7 ± 1.2 µg/m^3^ and P.ALP_0: 3.7 ± 2.1 µg/m^3^; P.ALP_1: 6.4 ± 2.1 µg/m^3^; P.ALP_2: 5.7 ± 2 µg/m^3^; P.ALP_3: 8.2 ± 2 µg/m^3^, respectively). Regarding the outdoor microenvironment, it can be stated that the P.ALPs generally overestimate the concentrations of PM_2.5_ when compared to the data acquired using the Aerocet device (P.ALP_0: −11 ± 6.1 µg/m^3^; P.ALP_1: −14 ± 5.1 µg/m^3^; P.ALP_2: −11 ± 7 µg/m^3^; P.ALP_3: 0.04 ± 4.6 µg/m^3^). These P.ALPs’ behaviors could be observed because different microenvironments are characterized by different PM concentrations with different characteristics (e.g., size distribution, hygroscopicity, and density) [31,32], which are known to influence the working principle (i.e., light scattering) of the PM sensor and, thus, the measurement performance [33]. Furthermore, as already demonstrated in a previous manuscript [22], by correcting the P.ALP data by following the same approach adopted for the Aerocet data (applying correction factors calculated using TWA HI’s data), the performances of the prototypes could significantly improve.

### 4.6. Overall Discussion on P.ALP Performance

The outcomes of the in-laboratory performance evaluation suggested that the P.ALP can follow PM_2.5_ concentration trend variations with reasonable efficacy, and it is characterized by a reliable performance regarding precision and accuracy [22]. Moreover, the outcomes of the present study regarding the P.ALP performances suggest the following:

(I) The precision between the four devices is good, and this performance does not change significantly when considering different concentration ranges and microenvironments.
(II) Concerning accuracy, the four prototypes are always comparable, but not mutually predictable, with the reference instrument (Aerocet). However, the P.ALP’s accuracy varies significantly among different CRs and microenvironments.
(III) Considering the whole dataset obtained from different testing conditions, the P.ALP is not suitable to be placed in one of the applicability tiers suggested by the US EPA. Nevertheless, after splitting the database based on CR and microenvironment, the P.ALP shows good performance, especially when investigating low and medium concentration ranges that characterize the tested office and outdoor microenvironments.
(IV) When dealing with extremely low concentrations of PM_2.5_, it was not possible to evaluate the P.ALP’s performance; conversely, at very high PM_2.5_ concentrations (occupational microenvironment), an overestimation trend was highlighted.
(V) It must be noted that all of the data presented in this study refer to raw measurements of the P.ALPs and, of course, it is possible to adopt correction or calibration factors to improve the accuracy of these devices.

As expected, in this study, it was highlighted that the P.ALPs’ performances may vary under different testing conditions. By following the “right sensor, used right” principle, it is possible to identify the P.ALP as a useful instrument for low and medium CRs for in-field applications (characteristic PM_2.5_ levels of urban backgrounds and office environments). Generally, as reported in Table S11 (Supplementary Materials) and summarized in Table 7, the P.ALP can be classified, at least, as an US EPA Tier I (education and information purposes) instrument for living environments.

Overall, the results of the field test carried out on the P.ALP in different concentration conditions and different microenvironments (Table 7 and Table S11) allow us to define, in general terms, the optimal scope of use of the P.ALP. The data obtained in this study suggest that it is possible to classify the P.ALP as a “Tier I” instrument according to the US EPA criteria [16] when applied in outdoor environments (ambient PM_2.5_ measurements) and in indoor air quality studies in residential or non-industrial work environments (e.g., offices, schools, etc.) when medium–low particulate concentrations are expected. It is worth noting that the P.ALP’s performance worsens at high concentrations and in industrial occupational environments. It is also worth noting that some specimens show better behaviors (Tier II: Hotspot Identification and Characterization; Tier IV: Personal Exposure) or worse behaviors (not classifiable according to US EPA criteria), suggesting that the P.ALP’s precision should be improved.

### 4.7. Strengths and Limitations of This Study

When evaluating the weaknesses of this study, it must be noted that this was the first in-field usage of P.ALP devices in the field and, at the time of the data acquisition process, they were still under development. Due to this confounder, malfunctions or technical problems resulted in the loss of some data, which, as a consequence, were not included in the analyzed database. Moreover, the device adopted as a reference (Aerocet), even if corrected, on average, by the data obtained through the gravimetrical analysis, could be affected by bias and measuring errors because it is not commonly considered as a reference-grade instrument itself. Finally, the data regarding the T and RH were collected during the whole study period. The latter data showed compatible conditions with the nominal functioning range of the P.ALP’s PM sensor (Plantower PMS5003; Working RH range 0~99%; working T range −10~60 °C). It is worth noting that both the T and RH are known interfering factors of light-scattering PM sensors [19]. Although it is expected that these interferences could typically occur at higher T and RH values than those observed in this study, the influences of T and RH on P.ALP’s performances in the observed working conditions will be further evaluated in future studies. Despite that, this project was the first to carry out an in-field assessment of the P.ALP prototype and, thanks to its careful study design, the authors were able to evaluate the device outside of laboratory conditions and deal with the real-world needs that only a field campaign can highlight. Furthermore, different microenvironments could be characterized by different dusts, and this may affect the performances of the devices depending on where they are used, as stated in the previous subsection.

### 4.8. Future Developments

Thanks to its low cost (<USD 150) and its open-source market positioning, the P.ALP might be adopted and further implemented by anyone interested in airborne pollutant monitoring. To better improve the device’s performance, especially in terms of data precision and accuracy, ad hoc post-correction factors could be produced, and dedicated calibrations, depending on the specific usage, might be performed. Lastly, the P.ALP device represents a solid base on which to implement a low-cost sensor network that could help to provide fundamental data on exposure trends with a high spatiotemporal resolution.

## 5. Conclusions

The development and availability of NGMS technologies contribute to the increase in the exposure of sciences to a higher level of interest by both citizens and the scientific community while allowing for the continual reduction in instrumentation costs. This fact inspired the authors of this manuscript to produce their own low-cost device that is able to monitor PM concentrations, T, and RH. Overall, it can be stated that the P.ALP prototype, in its current configuration, can follow PM_2.5_ concentration trends, and depending on the microenvironment in which it is adopted, it could generally be used for at least one of the US EPA-suggested application fields. As well documented in the scientific literature, the performances of these technologies in terms of data quality are generally not comparable with reference-grade devices but, once assessed, they could be used and adopted as tools that can help to achieve a specific goal. The novelty of this project is the “open source” approach that is adopted. All of the information regarding the construction, hardware, and software of the P.ALP is provided in a step-by-step guide (Fanti et al., 2023 [18]), which allows anyone who is interested in reproducing their own monitoring unit to do so. Since a novel prototype has been made available to anyone who wants to deal with these technologies and challenges, the authors are convinced that an in-field overall assessment of the performances must be conducted and provided. In conclusion, it must be underlined that this prototype should be validated with reference-grade instrumentation if the potential user is looking for reliable and accurate data, but it still is a useful device that is able to follow the temporal variability in PM concentrations, which is crucial in managing air pollution exposure.

## Figures and Tables

**Table 1:** Summary of data collecting sessions. Testing Day: number of monitoring days in field; ME: microenvironment investigated; Duration: minutes of monitoring per session (this number corresponds to N of valid data); PM_2.5_ Conc.: value of PM_2.5_ acquired using reference instrument (Aerocet), expressed in µg/m^3^; RH: relative humidity monitored during each session; T: average temperature monitored during each session.

Testing Day	ME	Duration [min]	PM_2.5_ [µg/m^3^]			RH [%]		T [°C]
			Min.	Mean	Max.	Min.	Max.	Mean
1	Office	480	3.4	6.3	9.1	28.1	30.4	22.7
2		480	0.3	1.2	3.9	18.9	23.7	21.4
3		480	2.1	4.4	6.8	27.1	29.3	22.6
4		480	4.9	6.7	8.9	32.3	37.0	22.8
5		480	1.9	3.9	18	32.4	34.6	22.7
6	Home	480	5.6	9.7	15	45.0	54.6	21.7
7		480	16	23	44	49.4	54.4	21.3
8		480	1.3	3.4	10	38.8	45.2	21.8
9		480	7.1	10	20	42.8	47.3	21.9
10		480	0.8	3.5	9.1	18.2	43.3	21.3
11	Outdoor	480	8.4	13	19	23.0	43.2	10.2
12		480	26	33	46	23.2	44.1	18.1
13		480	25	31	46	21.1	46.0	15.2
14		480	29	40	59	16.3	68.8	16.4
15		480	23	31	39	44.9	58.3	9.8
16	Occupational	480	307	502	622	35.4	38.5	19.7
17		360	170	383	566	38.4	46.4	18.1
18		360	71	297	437	22.4	32.3	17.9
19		360	73	301	481	19.1	29.3	19.7
20		360	62	291	364	31.1	33.8	20.2

The results of the statistical analyses conducted on the data acquired during the whole monitoring period are presented.

**Table 2:** PM_2.5_ concentrations acquired using different monitoring devices. Valid N: number of datapoints used for statistical analysis; Min.: minimum; Mean: mean value of data collected using the considered instrument; Median: median value of considered instrument; Max.: maximum; S.D.: standard deviation.

PM_2.5_—(µg/m^3^)						
Device	Valid N	Min.	Mean	Median	Max.	S.D.
Aerocet	9021	0.3	88	14	622	153
P.ALP_0	6584	2.2	198	41	982	289
P.ALP_1	5323	2.2	237	49	918	297
P.ALP_2	6614	2.2	198	42	929	293
P.ALP_3	4410	2.2	137	27	807	228

**Table 3:** Regression parameters between P.ALPs. R: Pearson correlation coefficient; R^2^: determination coefficient; q: intercept; m: slope; SE: standard error; C: comparable (following Watson et al.’s 1998 criteria [23]); MP: mutually predictable (following Watson et al.’s 1998 criteria [23]). The comparisons that satisfy Watson et al.’s criteria of comparability and/or mutual predictivity are highlighted in green.

Devices Compared	Regression Model					Watson et al.’s Criteria [23]	
	R	R^2^	q	m	SE	C	MP
P.ALP_0 vs. P.ALP_1	0.999	0.994	2.239	0.973	0.201	Yes	No
P.ALP_0 vs. P.ALP_2	0.999	0.999	−0.351	1.013	0.176	Yes	Yes
P.ALP_0 vs. P.ALP_3	0.999	0.997	−3.355	0.863	0.248	Yes	No
P.ALP_1 vs. P.ALP_2	1	0.999	−1.398	1.039	0.150	Yes	No
P.ALP_1 vs. P.ALP_3	0.999	0.998	−4.074	0.880	0.245	Yes	No
P.ALP_2 vs. P.ALP_3	0.999	0.998	−2.998	0.852	0.187	Yes	No

**Table 4:** The regression parameters between the four P.ALPs and the Aerocet. R: Pearson correlation coefficient; R^2^: determination coefficient; q: intercept; m: slope; SE: standard error; C: comparable (following Watson et al.’s 1998 criteria [23]); MP: mutually predictable (following Watson et al.’s 1998 criteria [23]). The comparisons that satisfy Watson et al.’s criteria of comparability and/or mutual predictivity are highlighted in green.

Devices Compared	Regression Model					Watson et al.’s Criteria [23]	
	R	R^2^	q	m	SE	C	MP
P.ALP_0 vs. Aerocet	0.956	0.914	3.187	1.634	1.290	Yes	Yes
P.ALP_1 vs. Aerocet	0.949	0.901	7.158	1.583	1.666	Yes	No
P.ALP_2 vs. Aerocet	0.958	0.917	1.018	1.662	1.279	Yes	Yes
P.ALP_3 vs. Aerocet	0.960	0.921	−9.789	1.562	1.173	Yes	No

**Table 5:** Application of US EPA Air Sensor Guidebook guidelines to place P.ALP prototypes in their application fields. Valid N: number of datapoints used for statistical analysis; Mean: mean of entire dataset utilized in this evaluation; SD: standard deviation; CV: coefficient of variation; CVdiff.: differential coefficient of variation between CV of reference-grade Aerocet instrument and the four different prototypes; MNB: mean normalized bias; Application Tier: result of application of US EPA criteria, in case of impossibility to categorize prototypes even in less stringent Tier (Tier I), “Failed” note was adopted.

Devices	PM_2.5_ [µg/m^3^]			US EPA Criteria			
	Valid N	Mean	SD	CV	CVdiff.	MNB	Application Tier
P.ALP_0	6584	198	289	1.46	−0.28	1.24	Failed
P.ALP_1	5323	237	298	1.26	−0.48	1.68	Failed
P.ALP_2	6614	199	293	1.48	−0.26	1.25	Failed
P.ALP_3	4410	138	228	1.65	−0.09	0.56	Failed
Aerocet	9021	88	154	1.74	-	-	-

**Table 6:** Statistics of average error trends used for Bland–Altman plots are presented in Figure 1. Mean: mean of entire dataset utilized in this evaluation; SD: standard deviation; Upper 95%: higher confidential interval in which top 95% of observations are included; Lower 95%: lower confidential interval in which bottom 95% of observations are included.

Devices Compared	PM_2.5_ Average Error [µg/m^3^]		PM_2.5_ Confidence Interval [µg/m^3^]	
	Mean	SD	Upper 95%	Lower 95%
Aerocet vs. P.ALP_0	−79	137	190	−347
Aerocet vs. P.ALP_1	−92	140	183	−367
Aerocet vs. P.ALP_2	−80	140	195	−355
Aerocet vs. P.ALP_3	−44	102	180	−243

**Table 7:** A summary of the application of the US EPA Air Sensor Guidebook guidelines, with the analysis split based on microenvironment and concentration range, to place the P.ALP prototypes in their application fields. ME: microenvironment investigated; CR: concentration range investigated, namely low concentrations (x < 6.69 µg/m^3^), medium concentrations (6.69 < x < 30.87 µg/m^3^), and high concentrations (x > 30.87 µg/m^3^). The “-” mark represents the combination of ME and CR that was not possible to investigate due to the unavailability of the data in that specific situation. For each combination of ME and CR, the results of the analysis of the four P.ALPs investigated are reported and highlighted in green. In the case of impossibility to categorize the prototypes, even in the less stringent tier (Tier I) suggested by the US EPA, the “Failed” note was adopted.

	ME	Low		Medium		High	
CR							
Office		Failed	I	II; IV	I	-	-
		II; IV	I	I	Failed	-	-
Home		-	-	II; IV	II; IV	-	-
		-	-	I	I	-	-
Outdoor		-	-	I	Failed	I	I
		-	-	I	III	I	V
Industrial		-	-	-	-	Failed	Failed
		-	-	-	-	Failed	I

## Data Availability

Data are contained within the article and Supplementary Materials.

## Supplementary Materials

The following supporting information can be downloaded at: https://www.mdpi.com/article/10.3390/toxics12040233/s1, Table S1: PM_2.5_ concentrations acquired with different monitoring devices; Table S2: PM_2.5_ concentrations acquired with different monitoring devices split by concentration range; Figure S1: Bar chart reporting the mean concentration values at the medium CR (6.96 < x < 30.87 µg/m^3^) expressed in [µg/m^3^] ± S.D. of the reference instrument (Aerocet) and the four P.ALP prototypes considering the set of data split by concentration ranges investigated. Figure S2: Bar chart reporting the mean concentration values at the high CR (>30.87 µg/m^3^) expressed in [µg/m^3^] ± S.D. of the reference instrument (Aerocet) and the four P.ALP prototypes considering the set of data split by concentration ranges investigated; Table S3: PM_2.5_ concentrations acquired with different monitoring devices split by microenvironment; Figure S3: Bar chart reporting the mean concentration values monitored, in the home ME, expressed in [µg/m^3^] ± S.D. of the reference instrument (Aerocet) and the four P.ALP prototypes considering the set of data split by ME; Figure S4: Bar chart reporting the mean concentration values monitored, in the outdoor ME, expressed in [µg/m^3^] ± S.D. of the reference instrument (Aerocet) and the four P.ALP prototypes considering the set of data split by ME; Figure S5: Bar chart reporting the mean concentration values monitored, in the occupational ME, expressed in [µg/m^3^] ± S.D. of the reference instrument (Aerocet) and the four P.ALP prototypes considering the set of data split by ME; Figure S6: Data distribution charts of the reference instrument (Aerocet) and of the four P.ALPs; Table S4: Regression parameters between P.ALPs splitting the dataset by concentration range; Table S5: Regression parameters between P.ALPs splitting the dataset by ME; Table S6: Regression parameters between the four P.ALPs and the Aerocet splitting the dataset by concentration range; Table S7: Regression parameters between the four P.ALPs and the Aerocet splitting the dataset by microenvironment; Table S8: Application of the EPA Air Sensor Guidebook guidelines to place the P.ALPs prototype in their application field splitting the dataset by concentration range; Table S9: Application of the EPA Air Sensor Guidebook guidelines to place the P.ALPs prototype in their application field splitting the dataset by microenvironment.; Table S10: Mann-Whitney test statistics; Table S11: Application of the EPA Air Sensor Guidebook guidelines to place the P.ALPs prototype in their application field splitting the dataset by microenvironment and concentration range; Figure S7: Bland-Altman plots of the four P.ALPs acquired data expressed in [µg/m^3^], focused on PM_2.5_ low concentrations, plotted against the reference instrument (Aerocet); Figure S8: Bland-Altman plots of the four P.ALPs acquired data expressed in [µg/m^3^], focused on PM_2.5_ medium concentrations, plotted against the reference instrument (Aerocet); Figure S9: Bland-Altman plots of the four P.ALPs acquired data expressed in [µg/m^3^], focused on PM_2.5_ high concentrations, plotted against the reference instrument (Aerocet).
