# Supplementary material for: An In-Field Assessment of the P.ALP Device in Four Different Real Working Conditions: A Performance Evaluation in Particulate Matter Monitoring
Source: Toxics. 2024 Mar 22;12(4):233. doi: 10.3390/toxics12040233 (PMC11054920; doi:10.3390/toxics12040233)
Supplement: Supplementary file 1 [file toxics-12-00233-s001.zip › toxics-2905489-supplementary.pdf]

Supplementary Materials

Article

An In-Field Assessment of The P.ALP Device in Four Different Real Working Conditions: A Performance Evaluation in Particulate Matter Monitoring

Giacomo Fanti [1,*], Francesca Borghi [2], Davide Campagnolo [1], Sabrina Rovelli [1], Alessio Carminati [1], Carolina Zellino [1], Andrea Cattaneo [1], Emanuele Cauda [3,4], Andrea Spinazzè [1] and Domenico Maria Cavallo [1]

1 Department of Science and High Technology, University of Insubria, 22100 Como, Italy; davide.campagnolo@uninsubria.it (D.C.); sabrina.rovelli@uninsubria.it (S.R.); acarminati@uninsubria.it (A.C.); czellino@uninsubria.it (C.Z.); andrea.cattaneo@uninsubria.it (A.C.); andrea.spinazze@uninsubria.it (A.S.); domenico.cavallo@uninsubria.it (D.M.C.)
2 Department of Medical and Surgical Sciences, University of Bologna, 40126 Bologna, Italy; francesca.borghi12@unibo.it
3 Center for Direct Reading and Sensor Technologies, National Institute for Occupational Safety and Health, Pittsburgh, PA 15236, USA; cuu5@cdc.gov
4 Centers for Disease Control and Prevention, Pittsburgh, PA 15236, USA
* Correspondence: giacomo.fanti@uninsubria.it; Tel.: +39-031-238-6645

Table S1. $PM_{2.5}$ concentrations acquired with different monitoring devices. Valid N: number of datapoint used for statistical analysis; Missing: number of missing values; <LOD: number of datapoints lower than the LOD of the considered instrument; Min.: minimum; Mean: mean value of the data collected by the considered instrument; Median: median value of the considered instrument; Max.: maximum; S.D.: standard deviation.

$PM_{2.5}$ – (μg/m³)								
**Device**	**Valid N**	**Missing**	**< LOD**	**Min.**	**Mean**	**Median**	**Max.**	**S.D.**
Aerocet	*9021*	*119*	*0*	*0.3*	*88*	*14*	*622*	*154*
P.ALP_0	7593	1547	1009	0	172	28	982	278
P.ALP_1	6607	2533	1284	0	191	42	918	283
P.ALP_2	8937	203	2323	0	147	14	929	267
P.ALP_3	7405	1735	2994	0	82	3.8	807	189

Table S2. $PM_{2.5}$ concentrations acquired with different monitoring devices split by concentration range. CR: concentration range, namely low concentrations (x < 6.69 μg/m³), medium concentrations (6.69 < x < 30.87 μg/m³) and high concentrations (x > 30.87 μg/m³); Valid N: number of datapoint used for statistical analysis; Min.: minimum; Mean: mean value of the data collected by the considered instrument; Median: median value of the considered instrument; Max.: maximum; S.D.: standard deviation.

PM2.5 – Dataset split by CR (µg/m³)							
**Device**	**CR**	**Valid N**	**Min.**	**Mean**	**Median**	**Max.**	**S.D.**
Aerocet	*Low concentration*	*2971*	*0.3*	*3.8*	*3.4*	*18*	*2*
	Medium concentration	*3000*	*7*	*17*	*14*	*44*	*8.5*
	High concentration	*3050*	*31*	*241*	*279*	*622*	*186*
P.ALP_0	Low concentration	793	2.2	7.9	5.6	44	6.3
	Medium concentration	2746	2.6	19	15	55	14
	High concentration	3045	29	409	469	982	314
P.ALP_1	Low concentration	499	2.2	3.6	3.4	6.1	0.8
	Medium concentration	1776	2.2	22	19	53	16
	High concentration	3048	27	401	465	918	304
P.ALP_2	Low concentration	787	2.2	3.4	3.2	6.7	0.9
	Medium concentration	2780	2.2	18	15	53	15
	High concentration	3047	27	414	476	929	317
P.ALP_3	Low concentration	300	2.2	2.9	2.8	4.5	0.6
	Medium concentration	2355	2.2	15	12	42	11
	High concentration	1755	19	326	301	807	268

Table S3. $PM_{2.5}$ concentrations acquired with different monitoring devices split by microenvironment. ME: microenvironment investigated; Valid N: number of datapoint used for statistical analysis; Min.: minimum; Mean: mean value of the data collected by the considered instrument; Median: median value of the considered instrument; Max.: maximum; S.D.: standard deviation.

PM2.5 – Dataset split by ME (µg/m³)							
**Device**	**ME**	**Valid N**	**Min.**	**Mean**	**Median**	**Max.**	**S.D.**
Aerocet	*Office*	*2374*	*0.3*	*4.5*	*5*	*18*	*2.4*
	Home	*2373*	*0.8*	*9.9*	*8.4*	*44*	*7.8*
	Outdoor	*2369*	*8.4*	*30*	*31*	*59*	*10*
	Occupational	*1905*	*62*	*364*	*343*	*622*	*123*
P.ALP_0	Office	844	2.2	7.8	5.7	44	6.1
	Home	1471	2.3	10	7.6	36	6
	Outdoor	2365	8.9	40	43	75	15
	Occupational	1904	114	623	675	982	186
P.ALP_1	Office	691	2.2	4	3.8	6.4	1
	Home	836	2.2	11	9.8	35	7.7
	Outdoor	1893	24	47	48	73	8.9
	Occupational	1903	116	610	662	918	175
P.ALP_2	Office	991	2.2	3.7	3.4	6.8	1.2
	Home	1353	2.2	8.9	4.8	40	7.5
	Outdoor	2367	6.8	41	45	66	15
	Occupational	1903	118	632	691	929	184
P.ALP_3	Office	502	2.2	3.2	3.1	4.7	0.7
	Home	947	2.2	8.8	6.7	32	6.5
	Outdoor	1893	4.3	27	30	50	11
	Occupational	1068	86	513	567	807	170

Table S4. Regression parameters between P.ALPs splitting the dataset by concentration range. CR: concentration range investigated, namely low concentrations ($x < 6.69$ µg/m³), medium concentrations ($6.69 < x < 30.87$ µg/m³) and high concentrations ($x > 30.87$ µg/m³); R: Pearson correlation coefficient; R^2: Determination coefficient; q: Intercept; m: Slope; SE: Standard Error; C: Comparable (following the Watson et al., 1998 criteria); MP: Mutually Predictable (following the Watson et al., 1998 criteria). CR "low concentration" was not evaluated due to the

poor numerosity of the data collected. In green are highlighted the comparisons that satisfy the Watson et al. criteria of comparability and/or mutual predictivity.

Devices Compared	CR	Regression Model					Watson et al. Criteria	
		R	R^2	q	m	SE	C	MP
P.ALP_0 vs. P.ALP_1	Medium concentration	0.988	0.976	-2.785	1.062	0.125	Yes	No
	High concentration	0.999	0.998	5.996	0.968	0.359	Yes	No
P.ALP_0 vs. P.ALP_2	Medium concentration	0.984	0.969	-2.466	1.055	0.091	Yes	No
	High concentration	0.999	0.998	2.842	1.008	0.427	Yes	No
P.ALP_0 vs. P.ALP_3	Medium concentration	0.963	0.928	-1.417	0.749	0.119	Yes	No
	High concentration	0.998	0.995	-1.543	0.86	0.718	Yes	Yes
P.ALP_1 vs. P.ALP_2	Medium concentration	0.994	0.988	0.759	0.977	0.072	Yes	No
	High concentration	0.999	0.999	-3.239	1.042	0.33	Yes	No
P.ALP_1 vs. P.ALP_3	Medium concentration	0.972	0.944	1.127	0.692	0.13	Yes	No
	High concentration	0.998	0.996	-6.431	0.884	0.617	Yes	No
P.ALP_2 vs. P.ALP_3	Medium concentration	0.984	0.968	0.002	0.721	0.068	Yes	No
	High concentration	0.998	0.996	-5.149	0.856	0.617	Yes	No

Table S5. Regression parameters between P.ALPs splitting the dataset by ME. ME: microenvironment investigated ; R: Pearson correlation coefficient; R^2: Determination coefficient; q: Intercept; m: Slope; SE: Standard Error; C: Comparable (following the Watson et al., 1998 criteria); MP: Mutually Predictable (following the Watson et al., 1998 criteria). ME "office" was not evaluated due to the poor numerosity of the data collected. In green are highlighted the comparisons that satisfy the Watson et al. criteria of comparability and/or mutual predictivity.

Devices Compared	ME	Regression Model					Watson et al. Criteria	
		R	R^2	q	m	SE	C	MP
P.ALP_0 vs. P.ALP_1	Home	0.972	0.945	5.656	0.896	0.514	Yes	No
	Outdoor	0.885	0.784	5.656	0.896	0.514	No	No
	Occupational	0.997	0.994	25.045	0.94	1.097	Yes	No
P.ALP_0 vs. P.ALP_2	Home	0.974	0.95	-4.124	1.208	0.094	Yes	No
	Outdoor	0.953	0.908	0.598	0.987	0.279	Yes	Yes
	Occupational	0.996	0.991	17.86	0.986	1.366	Yes	No
P.ALP_0 vs. P.ALP_3	Home	0.963	0.926	-4.515	1.036	0.137	Yes	No
	Outdoor	0.942	0.888	-1.632	0.752	0.25	Yes	No
	Occupational	0.992	0.993	25.915	0.819	2.055	Yes	No
P.ALP_1 vs. P.ALP_2	Home	0.98	0.977	0.148	1.062	0.076	Yes	Yes
	Outdoor	0.949	0.901	2.253	0.942	0.347	Yes	No
	Occupational	0.997	0.995	-7.232	1.048	1.135	Yes	No
P.ALP_1 vs. P.ALP_3	Home	0.97	0.942	-1.343	0.935	0.154	Yes	No
	Outdoor	0.873	0.761	-0.856	0.735	0.504	No	No
	Occupational	0.993	0.986	4.13	0.868	1.949	Yes	Yes
P.ALP_2 vs. P.ALP_3	Home	0.974	0.949	-0.741	0.837	0.088	Yes	No
	Outdoor	0.978	0.956	-1.153	0.746	0.15	Yes	No
	Occupational	0.993	0.986	3.79	0.843	1.981	Yes	Yes

Table S6. Regression parameters between the four P.ALPs and the Aerocet splitting the dataset by concentration range. CR: concentration range investigated, namely low concentrations, (medium concentrations and high concentrations; R: Pearson correlation coefficient; R^2: Determination coefficient; q: Intercept; m: Slope; SE: Standard Error; C: Comparable (following the Watson et al., 1998 criteria); MP: Mutually Predictable (following the Watson et al., 1998 criteria). In green are highlighted the comparisons that satisfy the Watson et al. criteria of comparability and/or mutual predictivity.

Devices Compared	CR	Regression Model	Watson et al. Criteria

		R	R^2	q	m	SE	C	MP
P.ALP_0 vs. Aerocet	Low concentration	0.452	0.205	12.009	-1.118	0.352	No	No
	Medium concentration	0.908	0.824	-7.236	1.507	0.261	Yes	No
	High concentration	0.92	0.847	33.344	1.557	3.677	Yes	No
P.ALP_1 vs. Aerocet	Low concentration	0.422	0.178	-0.827	0.717	0.427	No	No
	Medium concentration	0.898	0.807	-12.19	1.662	0.438	No	No
	High concentration	0.917	0.841	39.092	1.052	3.62	Yes	No
P.ALP_2 vs. Aerocet	Low concentration	0.27	0.073	1.821	0.256	0.201	No	No
	Medium concentration	0.913	0.833	-10.049	1.597	0.266	Yes	No
	High concentration	0.923	0.852	34.753	1.576	3.641	Yes	No
P.ALP_3 vs. Aerocet	Low concentration	0.248	0.62	2.264	0.098	0.148	No	No
	Medium concentration	0.913	0.833	-7.535	1.175	0.225	Yes	No
	High concentration	0.926	0.858	5.968	1.517	3.96	Yes	No

Table S7. Regression parameters between the four P.ALPs and the Aerocet splitting the dataset by microenvironment. ME: microenvironment investigated; R: Pearson correlation coefficient; R^2: Determination coefficient; q: Intercept; m: Slope; SE: Standard Error; C: Comparable (following the Watson et al., 1998 criteria); MP: Mutually Predictable (following the Watson et al., 1998 criteria). In green are highlighted the comparisons that satisfy the Watson et al. criteria of comparability and/or mutual predictivity.

Devices Compared	ME	Regression Model					Watson et al. Criteria	
		R	R^2	q	m	SE	C	MP
P.ALP_0 vs. Aerocet	Office	0.447	0.199	11.759	-0.986	0.334	No	No
	Home	0.962	0.925	-1.106	0.815	0.095	Yes	No
	Outdoor	0.943	0.89	0.272	1.357	0.309	Yes	Yes
	Occupational	0.681	0.463	250.451	1.027	9.772	No	No
P.ALP_1 vs. Aerocet	Office	0.688	0.474	-2.181	0.949	0.251	No	No
	Home	0.963	0.927	-5.735	0.961	0.176	Yes	No
	Outdoor	0.823	0.678	9.238	1.129	0.617	No	No
	Occupational	0.664	0.442	266.715	0.946	9.398	No	No
P.ALP_2 vs. Aerocet	Office	0.431	0.186	0.721	0.467	0.202	No	No
	Home	0.962	0.926	-5.818	1.009	0.127	Yes	No
	Outdoor	0.917	0.841	0.176	1.363	0.384	Yes	Yes
	Occupational	0.691	0.477	265.674	1.034	9.552	No	No
P.ALP_3 vs. Aerocet	Office	0.339	0.115	1.946	0.176	0.155	No	No
	Home	0.96	0.921	-6.161	0.88	0.156	Yes	No
	Outdoor	0.927	0.859	-5.235	1.194	0.316	Yes	No
	Occupational	0.702	0.493	142.382	1.141	12.139	No	No

Table S8. Application of the EPA Air Sensor Guidebook guidelines to place the P.ALPs prototype in their application field splitting the dataset by concentration range. CR: concentration range investigated, namely low concentrations, medium concentrations and high concentrations; Valid N: number of datapoint used for statistical analysis; Mean: mean of the entire dataset utilized in this evaluation; SD: standard deviation; CV: coefficient of variation; CV diff.: differential coefficient of variation between the CV of the reference grade instrument Aerocet and the four different prototypes. MNB: mean normalized bias; Application Tier: the result of the application of the EPA criteria highlighted in green, in case of impossibility to categorize the prototypes even in the less stringent tier (tier I) the "Failed" mark was adopted.

Devices	CR	$PM_{2.5}$ [μg/m³]			EPA criteria			
		Valid N	Mean	SD	CV	CVdiff.	MNB	Application Tier

P.ALP_0	Low concentration	793	7.9	6.3	0.80	0.26	1.10	Failed
	Medium concentration	2796	19	14	0.72	0.21	0.15	Tier II&IV
	High concentration	3045	409	314	0.77	0	0.69	Failed
P.ALP_1	Low concentration	499	3.6	0.8	0.22	-0.32	-0.04	Tier I
	Medium concentration	1776	22	16	0.73	0.23	0.32	Tier I
	High concentration	3048	401	304	0.76	-0.01	0.66	Failed
P.ALP_2	Low concentration	787	3.4	0.9	026	-0.27	-0.10	Tier II&IV
	Medium concentration	2780	18	15	0.83	0.32	0.07	Tier I
	High concentration	3047	414	317	0.76	-0.01	0.72	Failed
P.ALP_3	Low concentration	300	2.9	0.6	0.21	-0.33	-0.23	Tier I
	Medium concentration	2355	15	11	0.73	0.22	-0.13	Tier II&IV
	High concentration	1755	326	268	0.82	0.05	0.35	Tier I
Aerocet	Low concentration	2971	3.8	2	0.54	-	-	-
	Medium concentration	3000	17	8.5	0.51	-	-	-
	High concentration	3050	241	186	0.77	-	-	-

Table S9. Application of the EPA Air Sensor Guidebook guidelines to place the P.ALPs prototype in their application field splitting the dataset by microenvironment. ME: microenvironment investigated; Valid N: number of datapoint used for statistical analysis; Mean: mean of the entire dataset utilized in this evaluation; SD: standard deviation; CV: coefficient of variation; CVdiff.: differential coefficient of variation between the CV of the reference grade instrument Aerocet and the four different prototypes. MNB: mean normalized bias; Application Tier: the result of the application of the EPA criteria highlighted in green, in case of impossibility to categorize the prototypes even in the less stringent tier (tier I) the "Failed" note was adopted.

**Devices**	**ME**	**$PM_{2.5}$ [µg/m³]**			**EPA criteria**			
		**Valid N**	**Mean**	**SD**	**CV**	**CVdiff.**	**MNB**	**Application Tier**
P.ALP_0	Office	844	7.8	6.1	0.78	0.26	0.73	Failed
	Home	1471	10	6	0.58	-0.20	0.04	Tier II&IV
	Outdoor	2365	40	15	0.36	0.02	0.37	Tier I
	Occupational	1904	623	186	0.30	-0.04	0.71	Failed
P.ALP_1	Office	691	4	1	0.25	-0.27	-0.11	Tier II&IV
	Home	836	11	7.7	0.72	-0.06	0.08	Tier V
	Outdoor	1893	47	8.9	0.19	-0.15	0.60	Failed
	Occupational	1903	610	175	0.29	-0.05	0.68	Failed
P.ALP_2	Office	991	3.7	1.2	0.32	-0.20	-0.18	Tier II&IV
	Home	1353	8.9	7.5	0.84	0.06	-0.10	Tier III
	Outdoor	2367	41	15	0.37	0.03	0.37	Tier I
	Occupational	1903	632	184	0.29	-0.05	0.74	Failed
P.ALP_3	Office	502	3.2	0.7	0.22	-0.31	-0.29	Tier I
	Home	947	8.8	6.5	0.74	-0.04	-0.11	Tier III
	Outdoor	1893	27	11	0.43	0.08	-0.09	Tier V
	Occupational	1068	513	170	0.33	-0.01	0.41	Tier I
Aerocet	Office	2374	4.5	2.4	0.52	-	-	-
	Home	2373	9.9	7.8	0.78	-	-	-
	Outdoor	2369	30	10	0.34	-	-	-
	Occupational	1905	364	123	0.34	-	-	-

Table S10. Mann-Whitney test statistics; Z: Mann-Whitney test statistics; Asymp. Sig. significance.

**Devices Compared**	**Mann-Whitney U**	**Z**	**Asymp. Sig. (2-tailed)**
P.ALP_0 vs. Aerocet	20222613	-34.090	<0.05

P.ALP_1 vs. Aerocet	14541784	-39.516	<0.05
P.ALP_2 vs. Aerocet	22664454.5	-25.708	<0.05
P.ALP_3 vs. Aerocet	16992222	-13.738	<0.05

Table S11. Application of the EPA Air Sensor Guidebook guidelines to place the P.ALPs prototype in their application field splitting the dataset by microenvironment and concentration range. ME: microenvironment investigated; CR: concentration range investigated; Valid N: number of datapoint used for statistical analysis; Mean: mean of the entire dataset utilized in this evaluation; SD: standard deviation; CV: coefficient of variation; CVdiff.: differential coefficient of variation between the CV of the reference grade instrument (Aerocet) and the four different prototypes. MNB: mean normalized bias; "-": no data available for the analysis; Application Tier: the result of the application of the EPA criteria highlighted in green, in case of impossibility to categorize the prototypes even in the less stringent tier (tier I) the "Failed" note was adopted.

Devices Compared	ME	CR	$PM_{2.5}$ [µg/m^3]				EPA Criteria		
			Valid N	Mean	SD	CV	CVdiff.	MNB	Application Tier
P.ALP_0 VS Aerocet	Office	Low	692	8.30	6.60	0.80	0.25	1.07	Failed
		Medium	152	5.50	1.10	0.20	0.14	-0.26	Tier II&IV
		High	-	-	-	-	-	-	-
	Home	Low	-	-	-	-	-	-	-
		Medium	1370	10.70	6.00	0.56	0.07	-0.25	Tier II&IV
		High	-	-	-	-	-	-	-
	Outdoor	Low	-	-	-	-	-	-	-
		Medium	1224	30.70	12.60	0.41	0.07	0.38	Tier I
		High	1141	50.80	7.70	0.15	0.00	0.36	Tier I
	Occupational	Low	-	-	-	-	-	-	-
		Medium	-	-	-	-	-	-	-
		High	1904	622.90	186.00	0.30	-0.04	0.71	Failed
P.ALP_1 VS Aerocet	Office	Low	497	3.60	0.80	0.22	-0.32	-0.10	Tier I
		Medium	194	5.00	0.80	0.16	0.10	-0.33	Tier I
		High	-	-	-	-	-	-	-
	Home	Low	-	-	-	-	-	-	-
		Medium	834	10.70	7.70	0.72	0.23	-0.25	Tier II&IV
		High	-	-	-	-	-	-	-
	Outdoor	Low	-	-	-	-	-	-	-
		Medium	748	39.60	6.40	0.16	-0.18	0.77	Failed
		High	1145	52.30	6.30	0.12	-0.03	0.40	Tier I
	Occupational	Low	-	-	-	-	-	-	-
		Medium	-	-	-	-	-	-	-
		High	1903	610.30	175.40	0.29	-0.05	0.68	Failed
P.ALP_2 VS Aerocet	Office	Low	741	3.40	0.90	0.26	-0.28	-0.15	Tier II&IV
		Medium	250	4.50	1.30	0.29	0.23	-0.40	Tier I
		High	-	-	-	-	-	-	-
	Home	Low	-	-	-	-	-	-	-
		Medium	1307	9.20	7.50	0.82	0.33	-0.36	Tier I
		High	-	-	-	-	-	-	-
	Outdoor	Low	-	-	-	-	-	-	-
		Medium	1223	29.90	13.20	0.44	0.10	0.34	Tier I
		High	1144	51.70	6.00	0.12	-0.04	0.39	Tier I

	Occupational	Low	-	-	-	-	-	-	-
		Medium	-	-	-	-	-	-	-
		High	1903	632.10	184.20	0.29	-0.05	0.74	Failed
P.ALP_3 VS Aerocet	Office	Low	294	2.90	0.60	0.21	-0.34	-0.28	Tier I
		Medium	208	3.50	0.60	0.17	0.11	-0.53	Failed
		High	-	-	-	-	-	-	-
	Home	Low	-	-	-	-	-	-	-
		Medium	941	8.90	6.50	0.73	0.24	-0.38	Tier I
		High	-	-	-	-	-	-	-
	Outdoor	Low	-	-	-	-	-	-	-
		Medium	1206	21.00	10.00	0.48	0.13	-0.06	Tier III
		High	687	36.90	5.00	0.14	-0.02	-0.01	Tier V
	Occupational	Low	-	-	-	-	-	-	-
		Medium	-	-	-	-	-	-	-
		High	1068	512.70	170.30	0.33	-0.01	0.41	Tier I

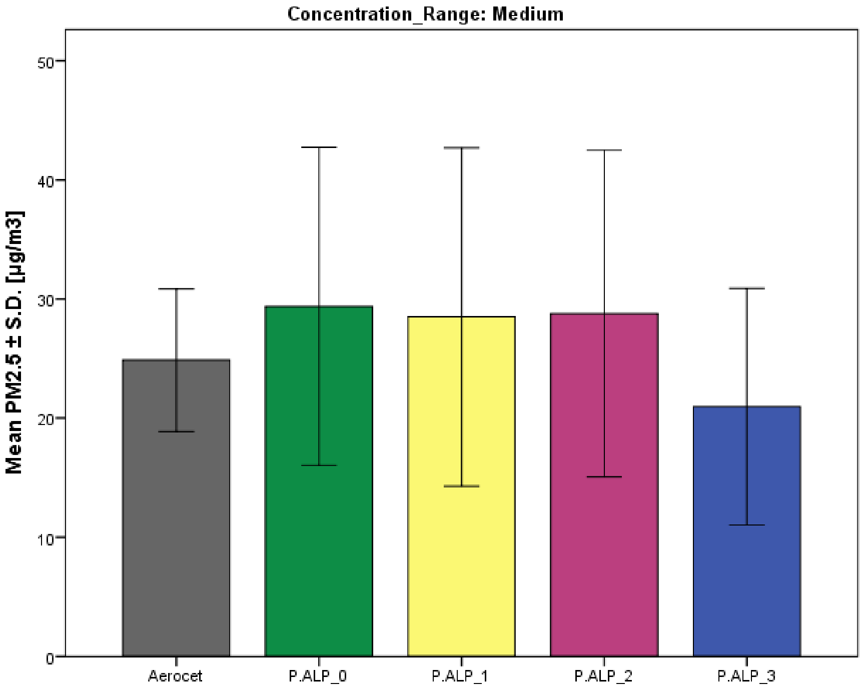

Figure S1. Bar chart reporting the mean concentration values at the medium CR (6.96 < x < 30.87 μg/m³) expressed in [μg/m³] ± S.D. of the reference instrument (Aerocet) and the four P.ALP prototypes considering the set of data split by concentration ranges investigated.

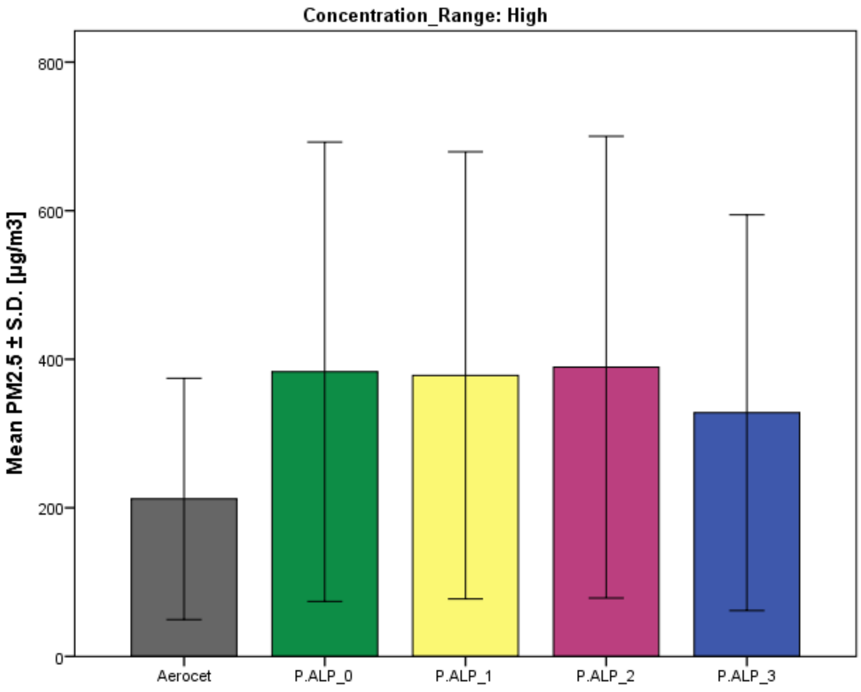

Figure S2. Bar chart reporting the mean concentration values at the high CR (> 30.87 μg/m^3) expressed in [μg/m^3] ± S.D. of the reference instrument (Aerocet) and the four P.ALP prototypes considering the set of data split by concentration ranges investigated.

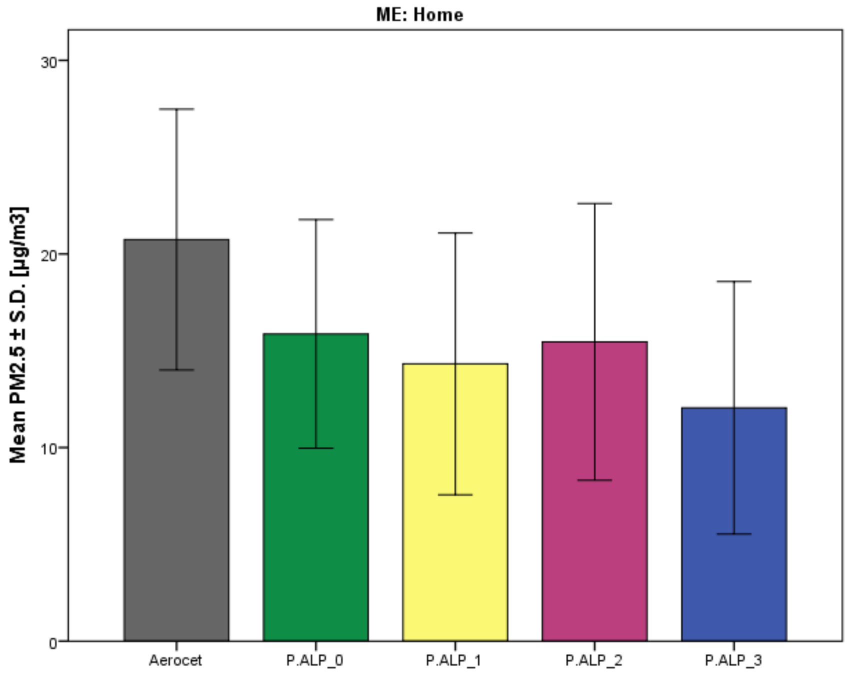

Figure S3. Bar chart reporting the mean concentration values monitored, in the home ME, expressed in [μg/m^3] ± S.D. of the reference instrument (Aerocet) and the four P.ALP prototypes considering the set of data split by ME.

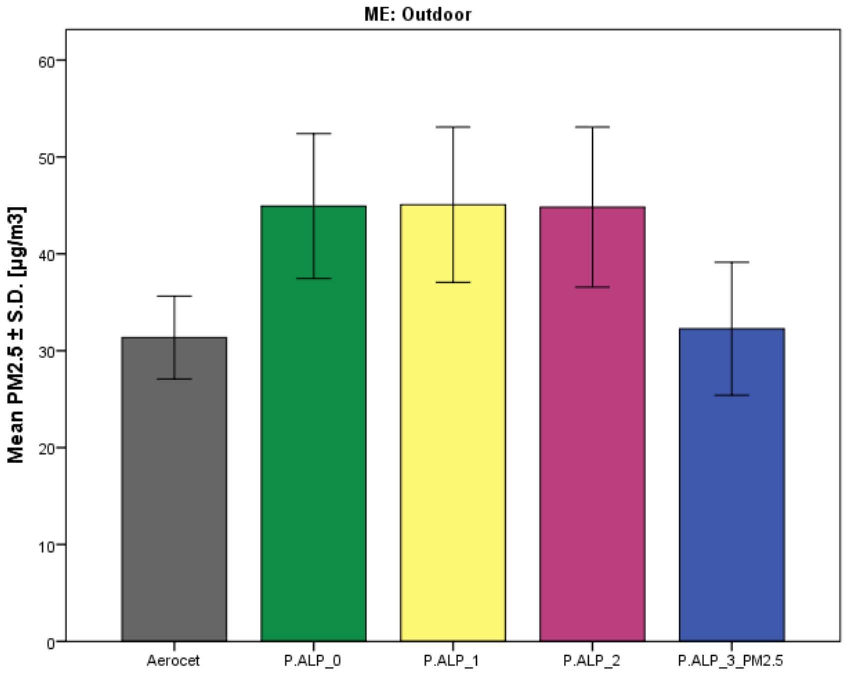

Figure S4. Bar chart reporting the mean concentration values monitored, in the outdoor ME, expressed in [μg/m^3] ± S.D. of the reference instrument (Aerocet) and the four P.ALP prototypes considering the set of data split by ME.

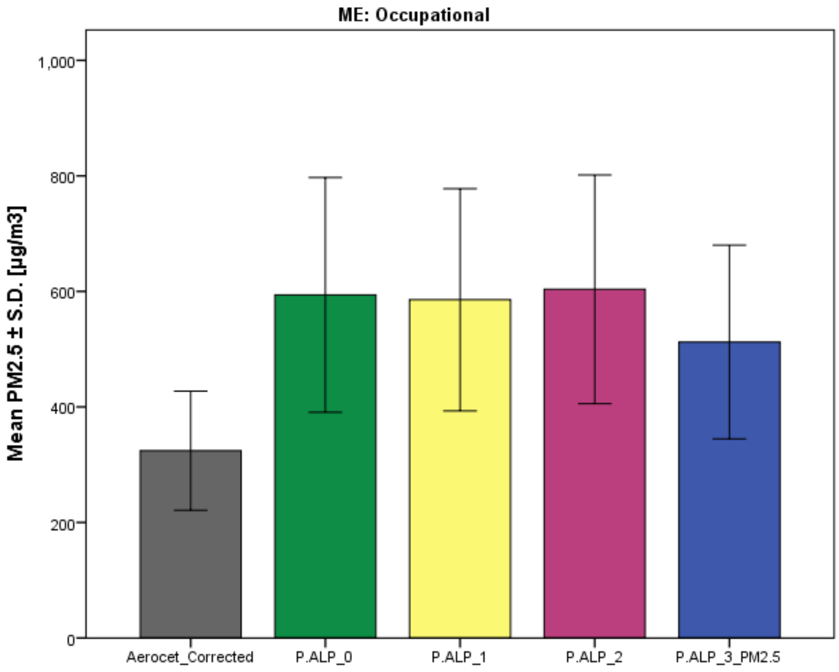

Figure S5. Bar chart reporting the mean concentration values monitored, in the occupational ME, expressed in [μg/m^3] ± S.D. of the reference instrument (Aerocet) and the four P.ALP prototypes considering the set of data split by ME.

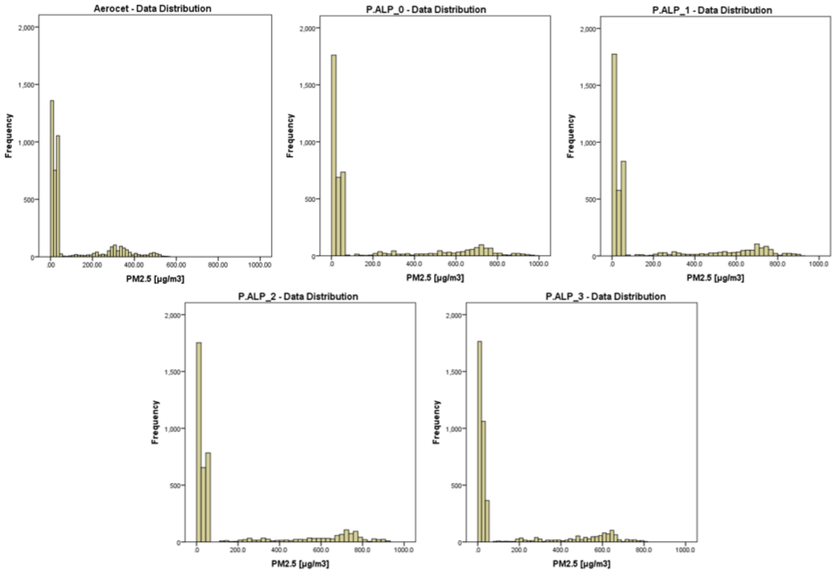

Figure S6. Data distribution charts of the reference instrument (Aerocet) and of the four P.ALPs.

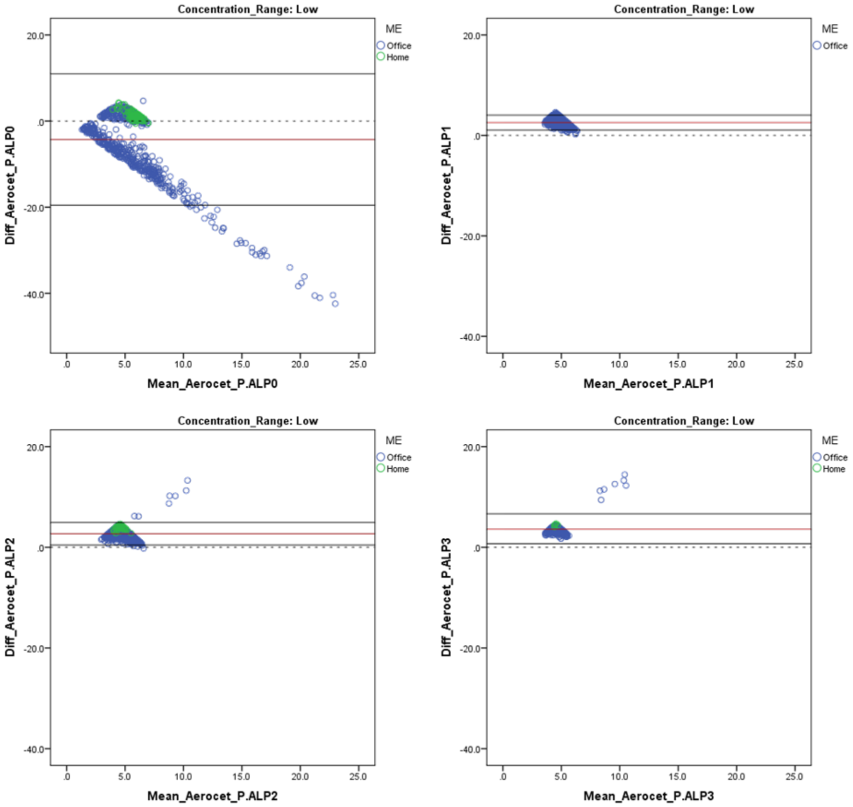

Figure S7. Bland-Altman plots of the four P.ALPs acquired data expressed in [µg/m^3], focused on $PM_{2.5}$ low concentrations, plotted against the reference instrument (Aerocet). In blue are highlighted the data referred to the Office ME and in green are highlighted the data referred to home ME. The dotted black line indicates the theoretic perfect agreement between the two compared instruments (P.ALP and Aerocet). The solid red line represents the mean error between the compared techniques and the two

solid black lines represent the upper and the lower 95% confidence intervals, respectively.

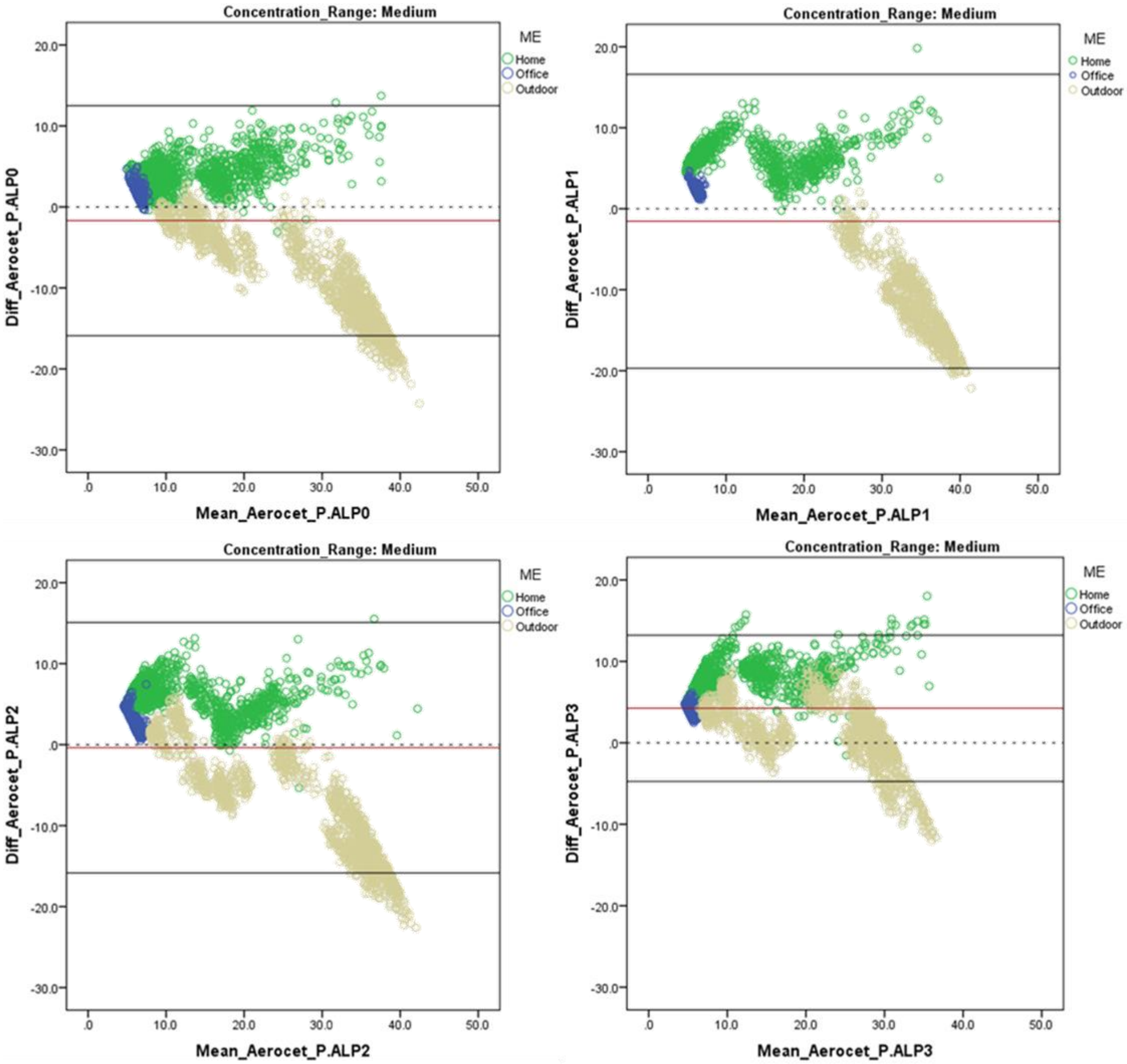

Figure S8. Bland-Altman plots of the four P.ALPs acquired data expressed in [μg/m^3], focused on $PM_{2.5}$ medium concentrations, plotted against the reference instrument (Aerocet). In blue are highlighted the data referred to the office ME and in green are highlighted the data referred to the home ME and in brown are highlighted the data referred to the outdoor ME. The dotted black line indicates the theoretic perfect agreement between the two compared instruments (P.ALP and Aerocet). The solid red line represents the mean error between the compared techniques and the two solid black lines represent the upper and the lower 95% confidence intervals, respectively.

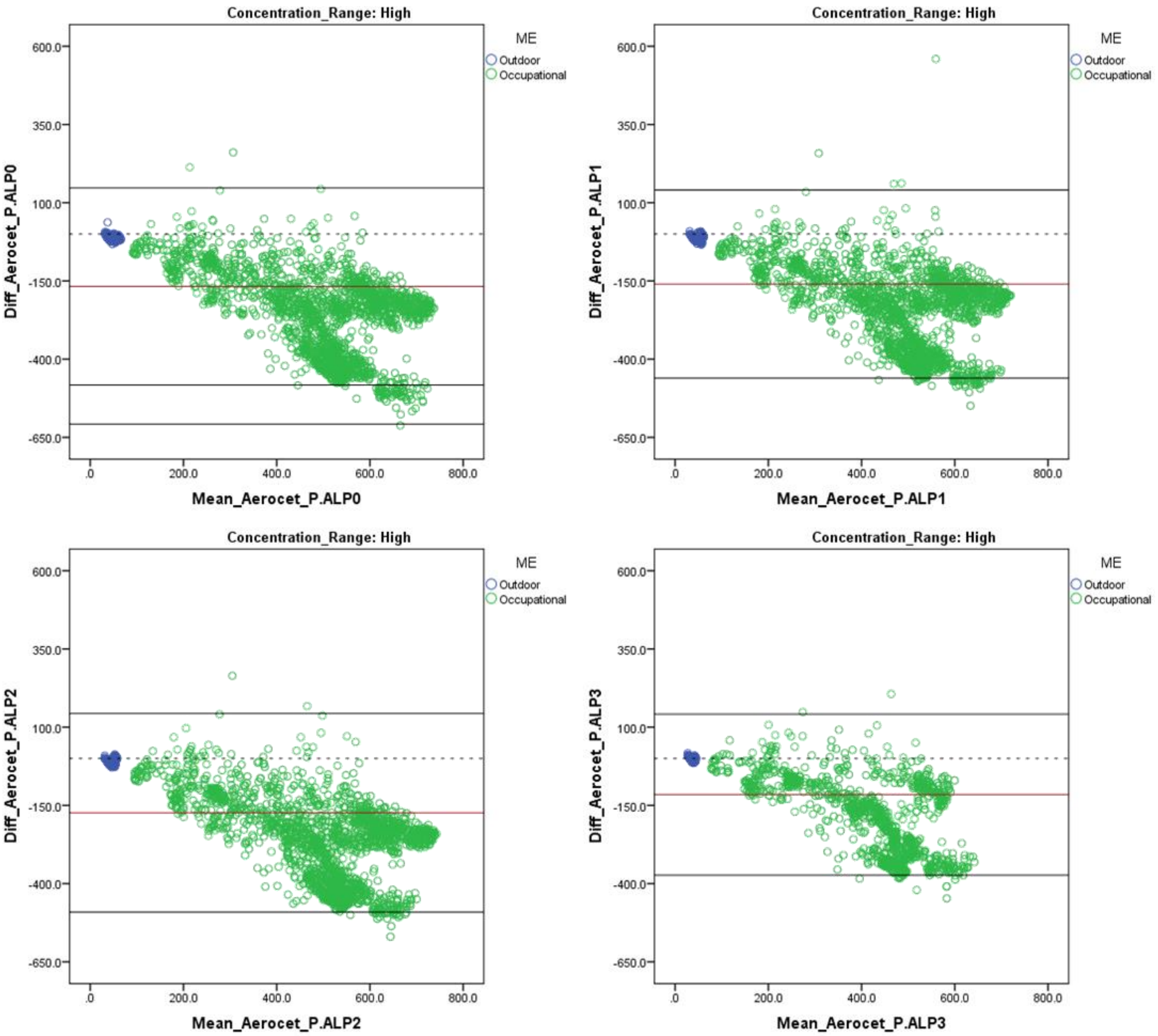

Figure S9. Bland-Altman plots of the four P.ALPs acquired data expressed in [μg/m^3], focused on $PM_{2.5}$ high concentrations, plotted against the reference instrument (Aerocet). In blue are highlighted the data referred to the outdoor ME and in green are highlighted the data referred to the occupational ME. The dotted black line indicates the theoretic perfect agreement between the two compared instruments (P.ALP and Aerocet). The solid red line represents the mean error between the compared techniques and the two solid black lines represent the upper and the lower 95% confidence intervals, respectively.
